# Corrigendum: The GIP Gamma-Tubulin Complex-Associated Proteins are Involved in Nuclear Architecture in *Arabidopsis Thaliana*

**DOI:** 10.3389/fpls.2020.589954

**Published:** 2020-09-11

**Authors:** Morgane Batzenschlager, Kinda Masoud, Natacha Janski, Guy Houlné, Etienne Herzog, Jean-Luc Evrard, Nicolas Baumberger, Mathieu Erhardt, Yves Nominé, Bruno Kieffer, Anne-Catherine Schmit, Marie-Edith Chabouté

**Affiliations:** ^1^Centre National de la Recherche Scientifique, Institut de Biologie Moléculaire des Plantes, UPR 2357, Conventionné avec l’Université de Strasbourg, Strasbourg, France; ^2^Biotechnologie et Signalisation cellulaire, Institut de Recherche de l’Ecole de Biotechnologie de Strasbourg, UMR 7242, Université de Strasbourg, Illkirch, France; ^3^Institut de Génétique et Biologie Moléculaire et Cellulaire, Ecole Supérieure de Biotechnologie de Strasbourg, Illkirch, France

**Keywords:** gamma-tubulin complex, AtGIP1/MOZART1, AtTSA1, nuclear envelope, *Arabidopsis thaliana*

## Errors in Supplementary Figures 3 and 4

In the original article, there were mistakes in **Supplementary Figures S3** **and** **S4** as published.

In the original article, there were mistakes in the **Supplementary Figure S3** ***as published*.**

Positive growth controls on –LW medium in the yeast two-hybrid growth assay.

Pictures of the AH109 cells co-transformed with recombinant vectors expressing proteins fused to the GAL4 Binding and Activating domains (BD and AD, respectively) were not properly selected (GIP1/TSA1-D1; GIP2/TSA1-D1; GIP1/TSA1-D4; GIP2/TSA1-D4; empty/TSA1-D1; empty/TSA1-D4; GIP1/empty; GIP2/empty).

Negative growth controls on –HLW + 3-AT medium.

Pictures of the AH109 cells co-transformed with recombinant vectors expressing proteins fused to the GAL4 Binding and Activating domains (BD and AD, respectively) were not properly selected (empty/TSA1-D1; empty/TSA1-D4; GIP1/empty; GIP2/empty; empty vectors).

Negative growth controls on –AHLW medium.

Pictures of the AH109 cells co-transformed with recombinant vectors expressing proteins fused to the GAL4 Binding and Activating domains (BD and AD, respectively) were not properly selected (empty/TSA1-D1; empty/TSA1-D4; GIP1/empty; GIP2/empty; empty vectors).

The corrected **Supplementary Figure S3** is now published.

In the original article, there were mistakes in the **Supplementary Figure S4** ***as published***:

Character inversion in the name of the pGBKT7 yeast two-hybrid vector

Negative control in the β-galactosidase filter assay.

Part of the picture showing the Y187 cells co-transformed with recombinant vectors pGAD10-GIP1 and pGBKT7 was not properly selected.

GIP1-GIP1 interaction in the β-galactosidase filter assay.

Part of the picture showing the Y187 cells co-transformed with recombinant vectors pGAD10-GIP1 and pGBKT7-GIP1 was not properly selected.

The corrected **Supplementary Figure S4** is now published.

The authors apologize for these errors and state that these do not change the scientific conclusions of the article in any way. The original article has been updated.

