# Supplementary figures and images for: Corrigendum: The GIP Gamma-Tubulin Complex-Associated Proteins are Involved in Nuclear Architecture in Arabidopsis Thaliana
Source: Front Plant Sci. 2020 Sep 11;11:589954. doi: 10.3389/fpls.2020.589954 (PMC7517941; doi:10.3389/fpls.2020.589954)

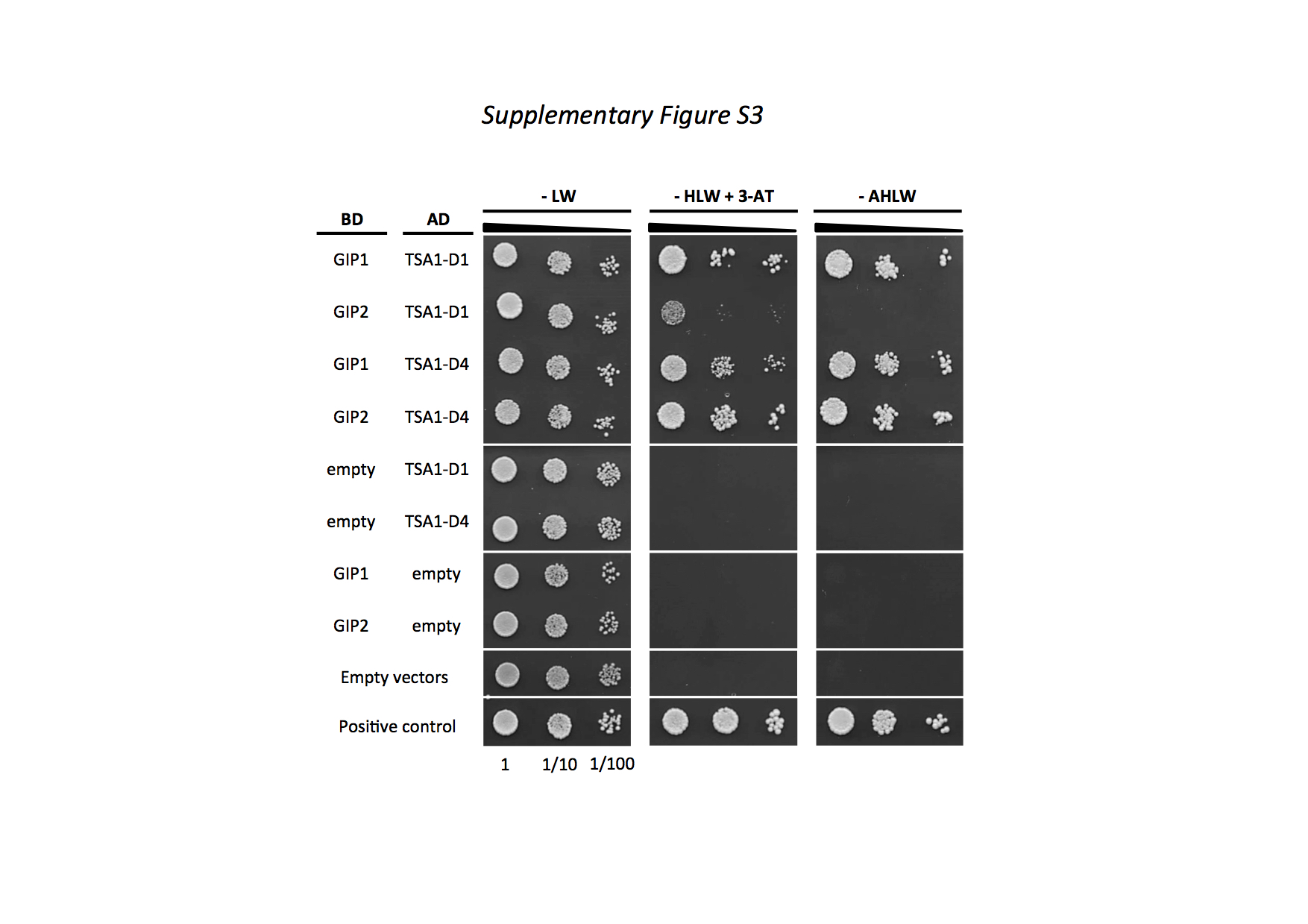

Supplement: Supplementary file 1 [file Image_1.jpeg]

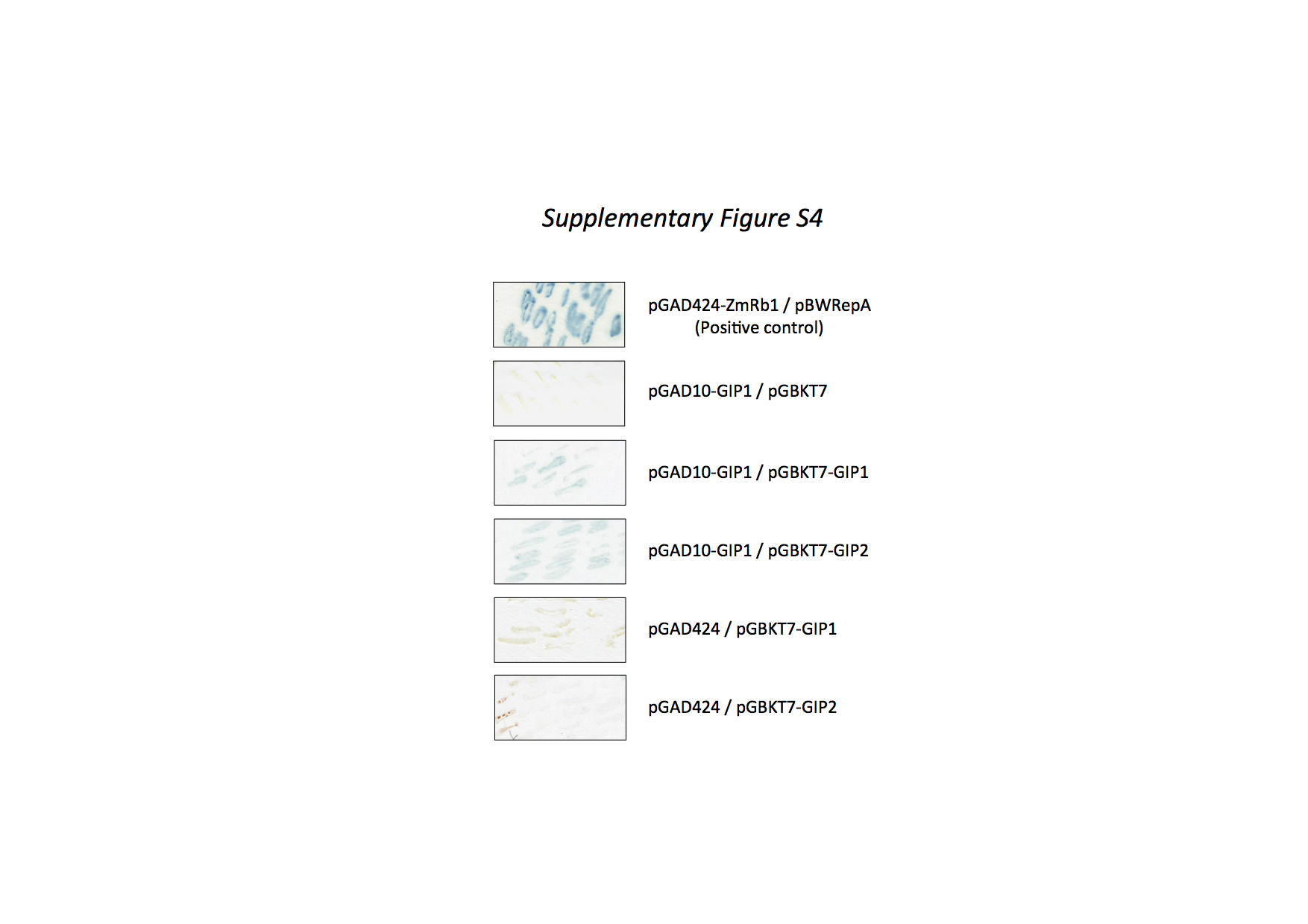

Supplement: Supplementary file 2 [file Image_2.jpeg]
